# Feasibility of Controlling Metastatic Osseous Pain Using Three Kinds of Image‐Guided Procedures for Thermal Microwave Ablation: A Retrospective Study

**DOI:** 10.1111/os.12849

**Published:** 2020-12-10

**Authors:** Jin Ke, Shi Cheng, Tao Yang, Chi Zhang, Ming Wang, Yu Zhang

**Affiliations:** ^1^ The Second School of Clinical Medicine Southern Medical University Guangzhou China; ^2^ Department of Orthopaedics Guangdong Provincial People's Hospital, Guangdong Academy of Medical Sciences Guangzhou China; ^3^ Department of Orthopaedics Guangdong Key Laboratory of Orthopaedic Technology and Implant Materials, General Hospital of Southern Theater Command Guangzhou China

**Keywords:** Microwave ablation, Osseous metastasis, Osseous pain

## Abstract

**Objectives:**

To evaluate the feasibility and safety of treating painful osseous metastases using image‐guided percutaneous thermal microwave ablation.

**Methods:**

This is a retrospective study of patients treated from December 2016 to December 2019 in one institute. A total of 50 patients (35 men, 15 women; mean age 55.24 ± 11.03 years) with 56 osseous metastatic lesions underwent image‐guided percutaneous microwave ablation. There were 7 patients with multiple and 43 patients with single metastases. The numbers of patients with primary cancer were as follows: lung, 13; liver, 17; kidney, 10; prostate, 1; breast, 3; osteosarcoma, 1; and thyroid, 5. Seventeen patients had cancer combined with soft tissue masses. The radiological images for the ablative procedures were obtained by CT, fluoroscopy with ultrasound, and fluoroscopy alone in 16, 11, and 23 patients, respectively. Pain severity was estimated using the visual analogue scale before and after treatment (1 week, 1 month, and 3 months after treatment). Radiological evaluations were performed at baseline and 3 months after the procedure.

**Results:**

In all patients, pain reduction occurred from the first day after treatment. Pain did not recur during the 3 months of follow up. The mean total ablation time per microwave electrode was 3.99 ± 2.48 min (range, 1–15 min). The mean power of the microwave electrode was 66.40 ± 12.08 W. The average volume of bone (load‐bearing bone, such as vertebra and acetabulum) cement after ablation was 2.82 ± 0.81 mL. There were no significant differences in visual analogue scale pain scores for different imaging techniques or ablation energies. No procedure‐related complications occurred.

**Conclusion:**

Image‐guided percutaneous thermal microwave ablation of osseous metastases relieves pain and improves mobility. CT remains the first choice for percutaneous ablation. Fluoroscopy combined with ultrasound is effective for cases with soft tissue masses; fluoroscopy is also suitable for combination with vertebroplasty. However, further investigations are required.

## Abstract 2

(A) Microwave ablation probe. (B) Temperature probe in the vertebral body and in the vertebral pedicle (parallel to the posterior margin). (C) Metastatic tumor. The thick white line of the ablation probe tip represents the source of microwave needle emission. The orange region represents the heating range of the microwave.

## Introduction

Bone metastases are common, painful complications in patients with advanced cancer^1^. Approximately 40% of cancers eventually develop bone metastases; the most common primary sources of these metastases are the breast, lung, and prostate^2^. These metastatic lesions can cause substantial oncologic pain: severe pain occurs in as many as 75% of patients with osseous metastases and can dramatically affect the quality of life^1^. Due to the short life expectancies and high systemic tumor burdens of affected patients^3^, therapeutic management focuses largely on palliative care and preservation of ambulatory functions rather than on curative strategies. The common goal of doctors and patients is to relieve metastatic cancer pain and improve survival and quality of life.

Management of osseous metastatic disease requires multidisciplinary input. In addition to continually evolving medical therapy regimens, surgical techniques, and radiation technologies, treatment options now include emerging minimally invasive interventions^4^. Treatment recommendations should be based on the collaborative recommendations of specialists, as outlined by the osseous Metastatic Disease Multidisciplinary Working Group, which has provided a consensus document detailing the evidence supporting their algorithms^5^, ^6^. Although radiation therapy has clearly demonstrated effective pain palliation in some patients, its benefit is not uniform, with 70%–80% of patients experiencing some pain relief and only 30% experiencing complete pain relief^7^. Furthermore, this improvement in pain requires several weeks to occur following radiation therapy and is frequently temporary, with pain returning in 57% of patients at a median of 15 weeks after radiation therapy^8^. With the progress of surgical techniques and the continuous development in the materials used for internal fixation, surgical treatment is playing an increasingly important role in the treatment of osseous metastatic carcinoma^9^. Moreover, in cases of osseous oligometastatic disease, extended resection may be performed with curative intent. Surgical treatment can either lead to effective resolution of intractable pain or neurological compromise and overt or impending spinal instability^10^, ^11^, ^12^. Surgical resection, reconstruction, or stabilization can be technically challenging in this potentially frail group of patients. As perioperative morbidity and prolonged recovery times may delay or interrupt systemic therapy, surgery is typically reserved for patients with a spinal cord compromise, existing pathologic fracture, or impending pathological fracture^13^. Generally, patients resort to analgesic drugs, which may be addictive^14^.

Due to these patients' short life expectancy and poor quality of life, a minimally invasive therapeutic approach is desirable. Recently, image‐guided ablation techniques have emerged and produced satisfactory results in the management of osseous metastatic pain^15^, ^16^, ^17^; such techniques include radiofrequency thermal ablation (RFA), microwaves (MW), laser ablation, magnetic resonance‐guided focused ultrasound surgery (MRgFUS), and cryoablation (CA), which uses extreme cold to destroy target tissues^18^. As a wide range of pain treatment options are available to patients with skeletal and soft‐tissue metastases, the selection of the most appropriate ablative technology requires proper patient and lesion selection, knowledge of relevant anatomy, and an understanding of the advantages and limitations of the specific technique^19^. Microwave ablation (MWA) is a newer heat‐based method that generates rapid heating of a large volume of tissue with decreased heat sink effects^20^. Due to the low conductivity of bony tissue, microwave energy should theoretically provide better tissue penetration than other types of thermal energy when treating osseous metastases^21^. In addition, multiple microwave antennas can be powered simultaneously to take advantage of the thermal synergy that occurs when these antennas are placed in close proximity^22^.

Khan *et al*. reported microwave ablation of 102 spinal metastases in 69 patients under CT or fluoroscopy. Follow‐up imaging at 20–24 weeks demonstrated no locoregional progression in 59 of the 61 surviving patients. Two complications were documented; one of them was S1 nerve thermal injury. Westbroek reported a case performed under fluoroscopy with the resultant complication of thermal injury to the spinal cord.

Kastler *et al*. reported successfully treating 20 spinal metastases (17 patients) with MWA (with cementoplasty in 9 cases). They reported pain relief in 16 of 17 patients, with significant pain reduction 1 day, 1 week, and 1, 3, and 6 months after ablation without any major complications. However, CT requires a large operational space, is time‐consuming, and has some radiation effects on patients and surgeons.

Ultrasound‐guided thermal surgery is now widely used in the management of lung, liver, and kidney cancers and has achieved satisfactory results. It provides real‐time visualization of applicator placement and intraoperative monitoring of the extent of ablation. However, it is not widely used in bone and soft tissue tumors and few reports exist of fluoroscopy combined with ultrasound‐guided microwave ablation^23^.

The purpose of our research was to answer the following three questions: i) whether using various image‐guided procedures (CT, fluoroscopy alone, and fluoroscopy combined with ultrasound) for microwave ablation in osseous metastases is safe; ii) whether using various image‐guided procedures is useful in controlling metastatic osseous pain; and iii) whether there are any differences between the three image‐guided percutaneous microwave ablation procedures for treating painful osseous metastases.

## Methods

All patients provided informed consent for participation and image publication, and were selected by an interdisciplinary team of radiation and medical oncologists and an orthopaedist (from the General Hospital of Southern Theater Command of PLA, Guangzhou, China), who performed image‐guided percutaneous MWA of osseous metastases between December 2016 and December 2019. This study was approved by the institutional review board. All procedures performed in this study were in accordance with the ethical standards of the institutional and/or national research committee and with the 1964 Helsinki declaration and its later amendments or comparable ethical standards.

Inclusion criteria were as follows: (i) patients who experienced refractory pain with a visual analogue scale (VAS) score of ≥4; (ii) lesions that did not respond to chemotherapy and/or radiotherapy; (iii) life expectancy >3 months; and (iv) ineligibility for surgical excision. Exclusion criteria were as follows: (i) active infections; (ii) lesions affecting key nerve roots or vascular bundles; and (iii) when the correlation between imaging findings and clinical symptoms was uncertain.

A total of 50 patients (35 men and 15 women; mean age, 55.24 ± 11.03 years) with 56 osseous metastatic lesions received image‐guided percutaneous MWA. All patients had previously received standard treatments: 39 received surgery on the primary site, 24 received chemotherapy, and 16 received chemotherapy and radiotherapy. There were 7 patients with multiple and 43 patients with single metastases. In addition, the numbers of patients with primary cancer were as follows: lung, 13; liver, 17; kidney, 10; prostate, 1; breast, 3; osteosarcoma, 1; and thyroid, 5. A total of 17 patients had cancer combined with soft tissue masses.

### *Procedure*

All patients underwent pre‐procedural contrast‐enhanced CT scanning and/or MRI to evaluate the size and location of and the relationships among lesions, nerves, and vascular bundles and to determine the ideal location of the antenna and scope of MWA.

Ablation was performed with an MWA system (2450 MHz, MTI‐5A, Great Wall, Nanjing, China). CT‐guided surgery, fluoroscopy combined with ultrasound, and fluoroscopy were performed for 16, 11, and 23 cases, respectively. The choice of guidance with CT, tomography (X‐rays), or ultrasound with tomography depended on the type of lesions and associated soft tissue masses. Throughout the procedure, 12 patients were consciously sedated using continuous intravenous infusion of fentanyl citrate (0.1 mg/2 mL diluted to 1:10 with saline solution) and received local anesthesia composed of a subcutaneous injection of 2% lidocaine hydrochloride^24^. A total of 24 patients received local anesthesia, 13 patients general anesthesia, and 1 patient epidural anesthesia. All treatments were executed by the same surgeon, with the cooperation of an ultrasonographer as required.

**Fig. 1:**
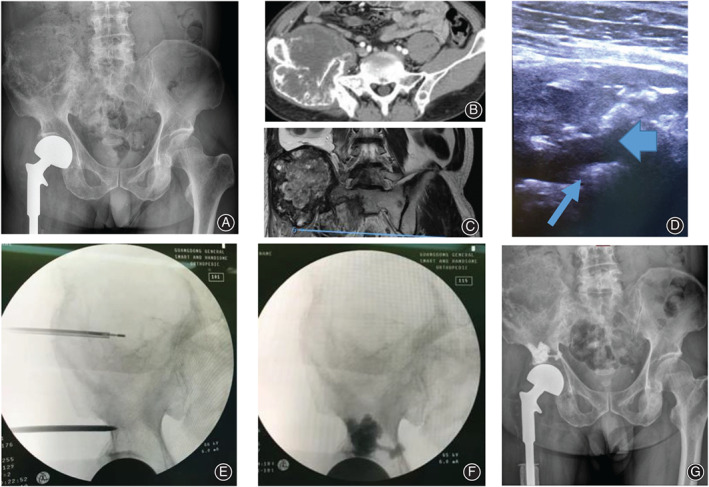
(A) A 59‐year‐old patient with lung cancer who was previously treated with hip prosthesis reconstruction for multiple hip metastases, including metastasis to the right acetabulum. (B, C) CT and MRI show osteolytic metastases with a large soft tissue mass in the right iliac bone. (D) This case underwent percutaneous microwave ablation guided with X‐ray combined with ultrasound. The ablation zone during microwave ablation (thin arrow), an osteolytic zone with soft tissue mass (thick arrow), which were shown on ultrasound. (E) The microwave probe guided by fluoroscopy, With the aid of ultrasound, the microwave needle was located in the lesion and adjusetd the Angle and depth of the needle gradually. (F) After the ablation, cementoplasty was performed guided with fluoroscopy. (G) The postoperative fluoroscopy showed no leakage of bone cement.

**Fig. 2:**
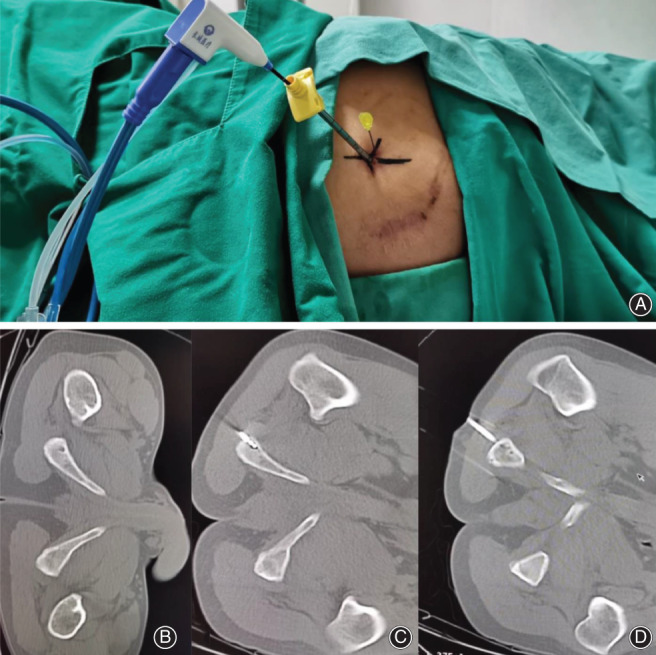
(A) A 37‐year‐old man with liver cancer who received CT fluoroscopy‐guided percutaneous microwave ablation for the treatment of the ischial tuberosity ilium metastasis. (B, C, D) The microwave probe guided by CT and the thermocouple needle is situated in the surrounding normal tissue.

**Fig. 3:**
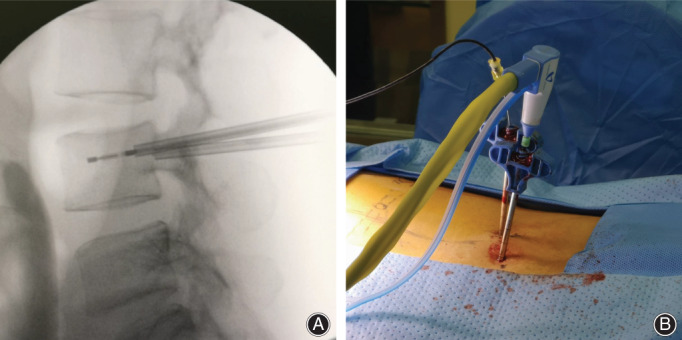
(A and B) A 44‐year‐old woman with lung cancer who received fluoroscopy‐guided percutaneous microwave ablation for the treatment of third lumbar vertebral metastasis.

An 11‐gauge bone biopsy needle was inserted into the proximal edge of the lesion under image guidance. The choice of ultrasonic probe was adjusted according to the depth of the lesion: a high frequency linear ultrasound probe was selected for the detection of superficial sites, including superficial masses and blood vessels, while a convex array transducer was used for deep tumors and deep blood vessels (such as the external iliac artery). Subsequently, the needle shaft was withdrawn, and the biopsy needle was advanced into the lesion. Once the appropriate position was confirmed, core biopsy samples were obtained. A 15‐gauge or 14‐gauge MWA antenna (diameter: 1.8 mm, length: 180 mm) was coaxially inserted into the tumor until the tip of the antenna was adjacent to the edge of the lesion. Technical success was defined as the ability to place the antenna accurately into the tumor and to calculate the ablation zone 2–3 mm beyond the extent of the tumor in an oval configuration; if the distance from a neurovascular bundle was <5 mm, the above requirements were not adhered to. Concurrently with ablation, a thermometer needle was placed in the normal tissue to ensure that the temperature remained below 43°C. Because the temperature probe only reflected the temperature at its tip, two to three probes were placed simultaneously to monitor the temperature at different sites, such as deeper intraoperative positions. After ablation, the antenna was withdrawn, and, if required, the introducer was left *in situ* for the osteoplastic procedure. Illustrative cases of each image‐guided procedure are shown in Figs 1, 2, 3. Cementation was used for the weight‐bearing bones, such as the acetabulum (*n* = 1) and vertebrae (*n* = 24). The amount of bone cement injected under image guidance was determined based on tumor size. In the case illustrated in Fig. 1, the cement volume was low because of the incomplete acetabular wall. However, the acetabular bearing region was well filled. The mean volume injected per tumor was 2.82 ± 0.81 mL. Post‐procedural fluoroscopy or CT determined whether any complications, such as bleeding or bone cement leakage, occurred.

### *Evaluation Index*

Before the ablation treatment and at follow up, the VAS was used to evaluate the pain severity. Pain intensity was rated on a continuous scale from 0 to 10^25^. Functional outcome was evaluated using a qualitative scale for the assessment of the patient's walking ability and was rated as worse, unchanged, or improved^24^. Basic data regarding operation‐related parameters were recorded, and no complications were observed. Table 1 summarizes the baseline characteristics of all patients.

**TABLE 1:** Classification of osseous metastasis with regard to primary tumors and site of metastasis

Primary tumor	*n*	Site of metastasis	*n*
Lung	13	Spine	24
Liver	17	Rib	6
Kidney	10	Acetabulum	1
Breast	3	Ilium	15
Prostate	1	Ischial tuberosity	2
Osteosarcoma	1	Clavicle	1
Thyroid	5		

### *Visual Analogue Scale*

Continuous data for pain intensity was recorded using the VAS. The score was determined by measuring the distance (mm) on the 10‐cm line between the “no pain” anchor and the patient's mark using a ruler, providing a range of scores from 0 to 10; a higher score indicated a greater pain intensity. The following cut‐off points pain using the VAS have been recommended: no pain, 0; mild pain, ≤3; moderate pain, 4–6; and severe pain, 7–10^26^.

### *Statistical Analysis*

SPSS 24.0 (SPSS, Chicago, IL, USA) was used for data analyses. Continuous variables are shown as mean ± standard deviation. Student's *t*‐test was applied to evaluate VAS scores before and after the procedure; *P* < 0.05 was considered statistically significant.

## Results

### *Patient Demographics and Surgical Data*

A total of 35 men and 15 women were included in this study. The mean total ablation time per MW electrode was 3.99 ± 2.48 min (range: 1–15 min). The mean power of the MW was 66.40 ± 12.08 W (range: 40–80 W). Approximately 40% (20/50) of cases (25 sites) underwent the osteoplastic procedure; the mean volume of bone cement injected was 2.82 ± 0.81 mL.

### *Postoperative Evaluation*

Based on the results obtained, the three questions posed by this study were answered in the following manner.

### *Complications*

**Fig. 4:**
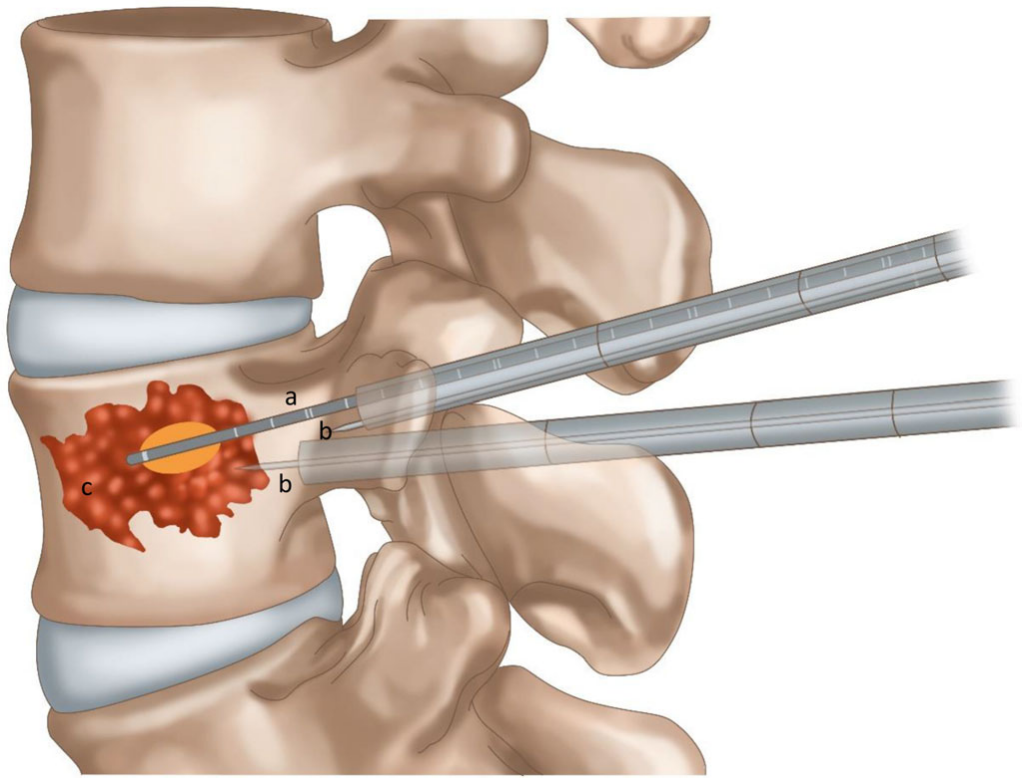
(A) Microwave ablation probe. (B) Temperature probe in the vertebral body and in the vertebral pedicle (parallel to the posterior margin). (C) Metastatic tumor. The thick white line of the ablation probe tip represents the source of microwave needle emission. The orange region represents the heating range of the microwave.

**Fig. 5:**
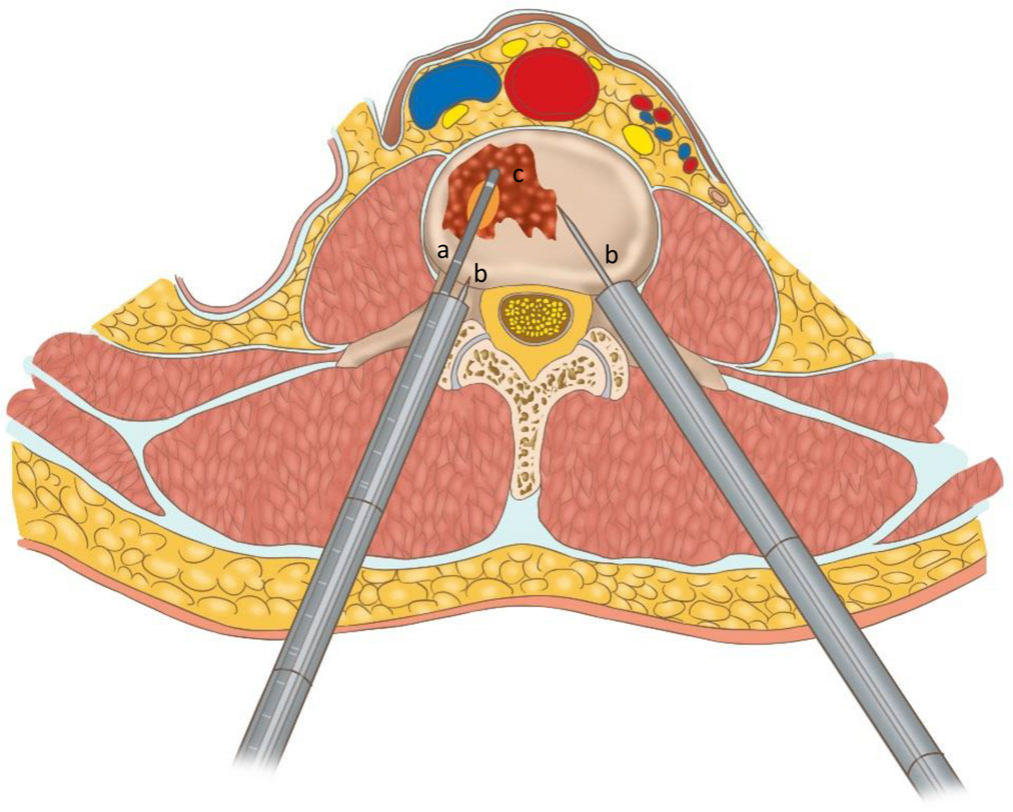
(A) Microwave ablation probe. (B) Temperature probe in the vertebral body and in the vertebral pedicle (parallel to the posterior margin). (C) Metastatic tumor. The thick white line of the ablation probe tip represents the source of microwave needle emission. The orange region represents the heating range of the microwave.

**Fig. 6:**
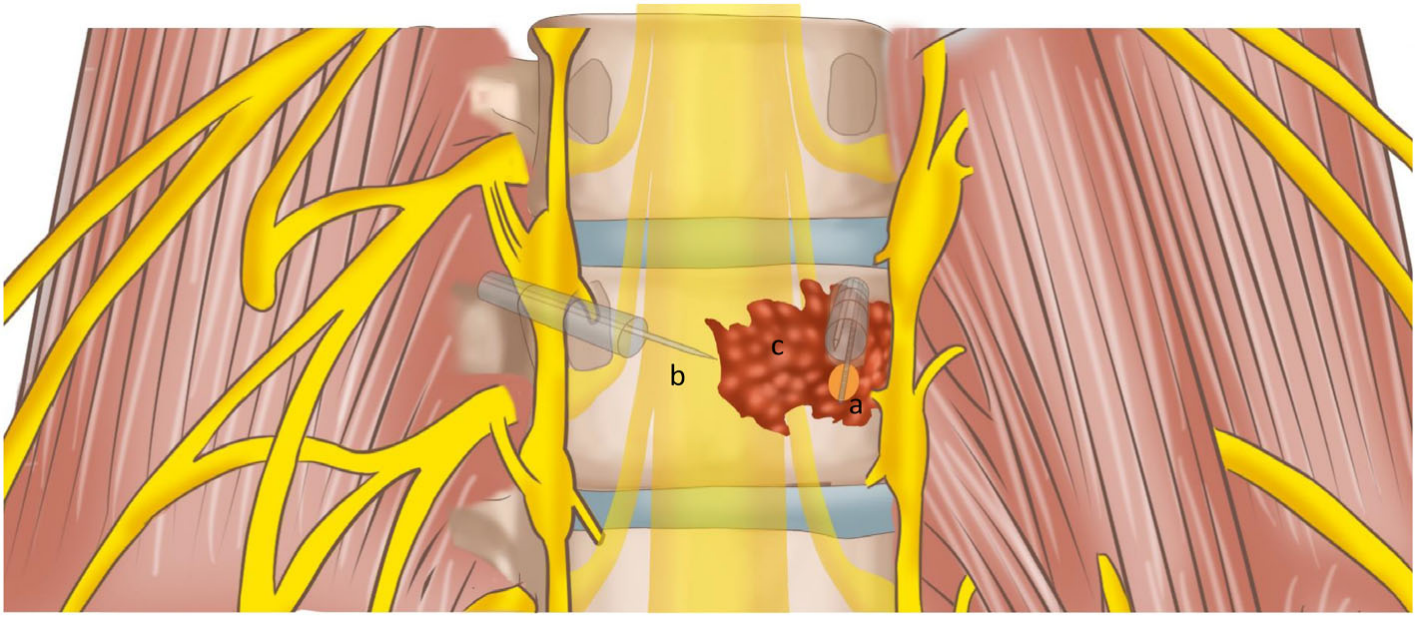
(A) Microwave ablation probe. (B) Temperature probe in the vertebral body and in the vertebral pedicle (parallel to the posterior margin). (C) Metastatic tumor. The thick white line of the ablation probe tip represents the source of microwave needle emission. The orange region represents the heating range of the microwave.

(1) Whether using various image‐guided procedures (CT, fluoroscopy alone, and fluoroscopy combined with ultrasound) for microwave ablation in osseous metastases is safe: None of the patients had complications related to the three image‐guided procedures, such as neurovascular bundle injury. Two patients who underwent ablation procedures assisted by fluoroscopy combined with ultrasound developed symptoms of fever on postoperative days 1 and 3 that lasted for 24–48 h. This was attributed to post‐ablation syndrome. Illustrative emulation of intraoperative ablation and monitoring is shown in Figs 4, 5, 6.

### *Pain*

**Fig. 7:**
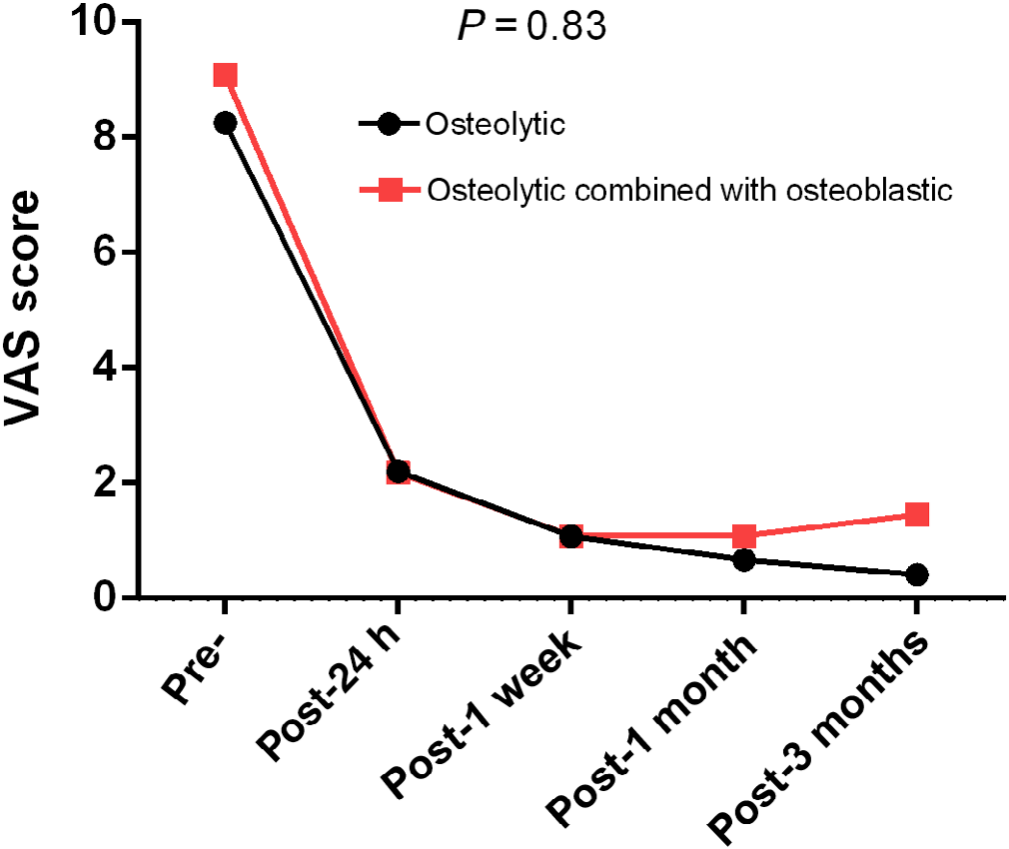
Changes in visual analogue scores from baseline to 3 months between osteolytic and osteolytic combined with osteoblastic groups.

(2) Whether using various image‐guided procedures is useful in controlling metastatic osseous pain: VAS scores for the assessment of pain were recorded preoperatively and postoperatively (24 h, 1 week, 1 month, and 3 months after the procedure). All patients achieved satisfactory pain relief. The preoperative VAS scores ranged from 6 to 10, with an average of 8.75 ± 1.03 before CT‐guided operation, 8.33 ± 1.50 before fluoroscopy combined with ultrasound, and 8.66 ± 1.41 before fluoroscopy. Average VAS scores 24 h after ablation procedures guided by these imaging modalities were 2.37 ± 1.08, 2.00 ± 1.26, and 2.17 ± 1.19, respectively. The mean reductions in VAS scores were calculated from 1 week to 3 months after the procedure. The postoperative VAS scores for lung, liver, prostate, breast, thyroid, kidney, and bone metastases were significantly lower than the preoperative scores (preoperative: 8.76 ± 1.01, 8.35 ± 1.27, 10.00 ± 0.00, 9.33 ± 1.15, 6.80 ± 1.09, 8.40 ± 1.26, 10.00 ± 0.00, respectively; postoperative [24 h]: 2.30 ± 1.10, 1.88 ± 1.11, 4.00 ± 0.00, 3.33 ± 1.15, 2.80 ± 1.09, 1.80 ± 1.13, 2.00 ± 0.00, respectively). No significant differences in VAS scores were observed among the different primary lesions. The lesions were divided into osteolytic and osteolytic combined with osteoblastic groups through imaging analysis, such as X ray, CT, and MRI. Although patients in the osteolytic group had better postoperative pain improvement, there was no statistical significance in the changes of VAS curves between the two groups. The VAS score curves are shown in Fig. 7.

### *Comparison of the Three Image Modes*

**Fig. 8:**
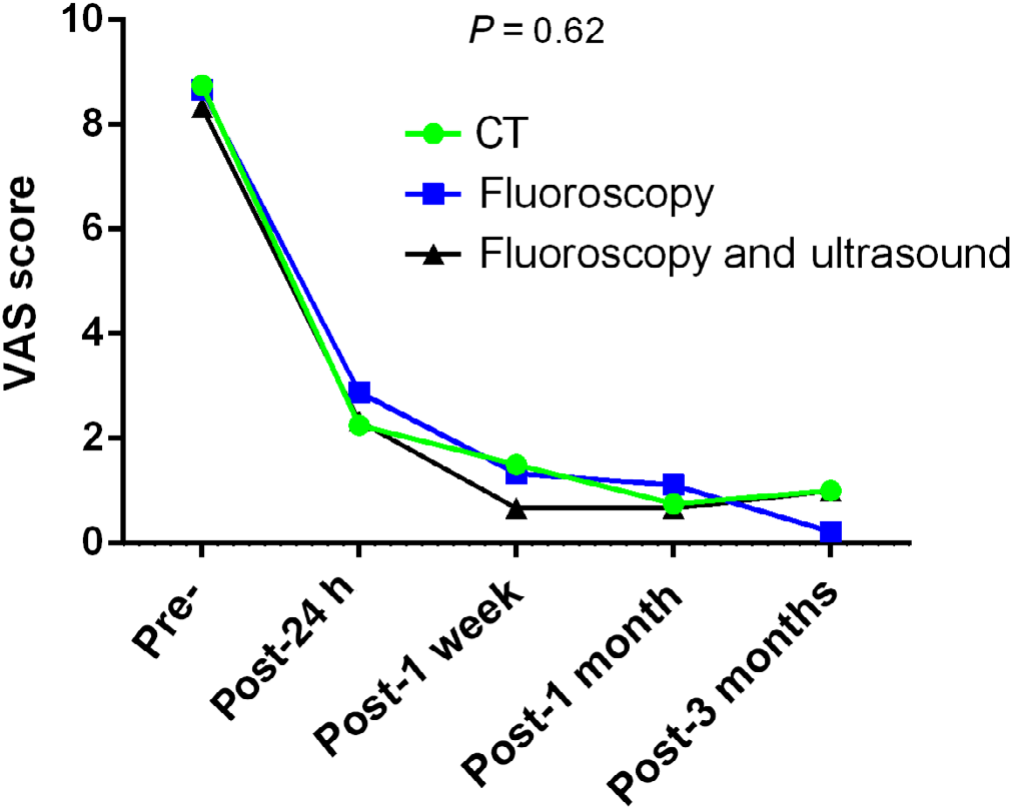
Changes in visual analogue scores from baseline to 3 months after various image‐guided percutaneous thermal microwave ablations for osseous metastasis.

**Fig. 9:**
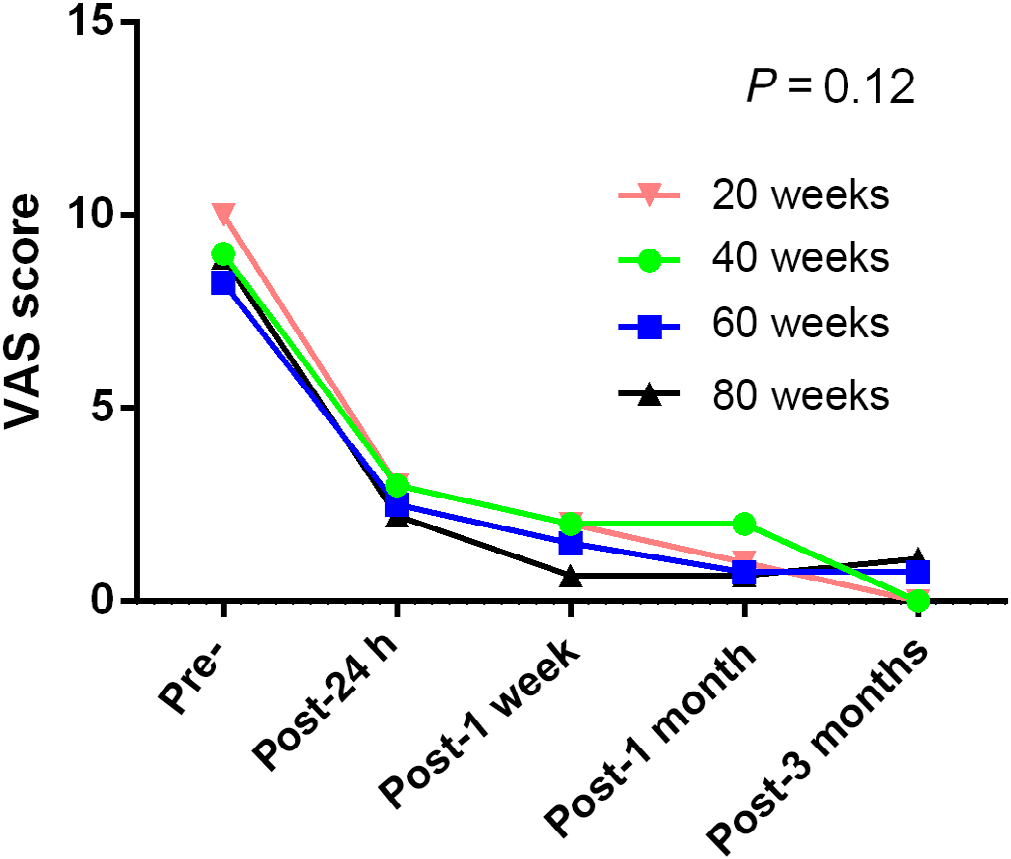
Changes in visual analogue scores from baseline to 3 months *versus* ablation energy.

**Fig. 10:**
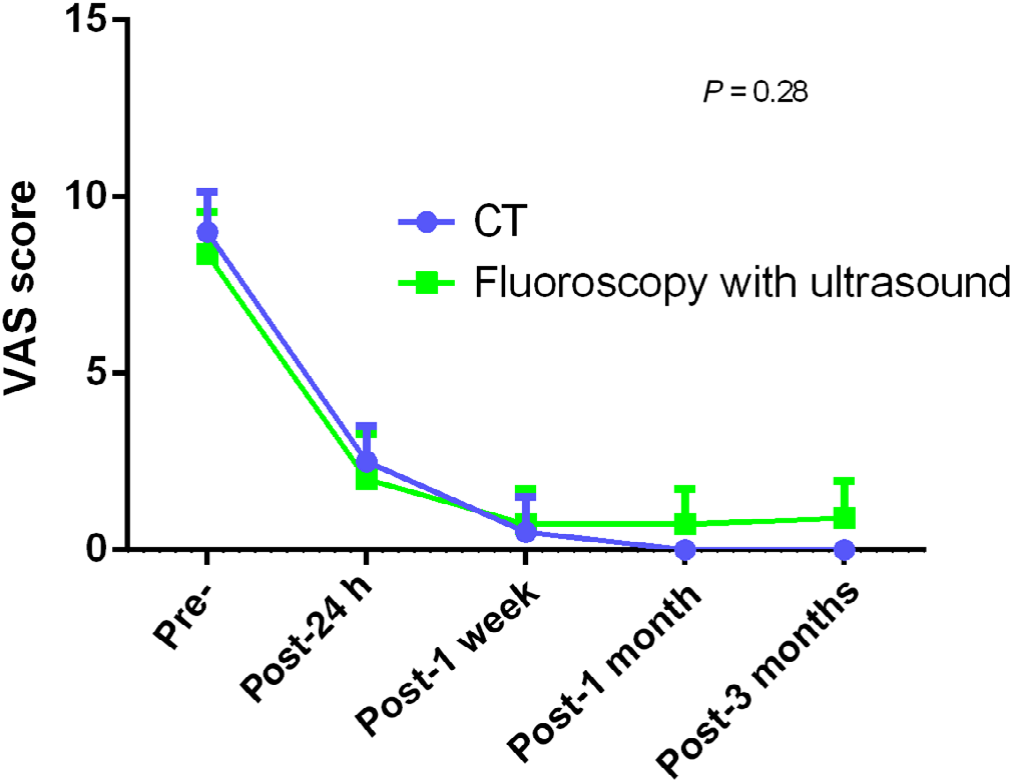
Changes in visual analogue scale scores from baseline to 3 months between CT and fluoroscopy with ultrasound in patients with soft‐tissue masses.

**Fig. 11:**
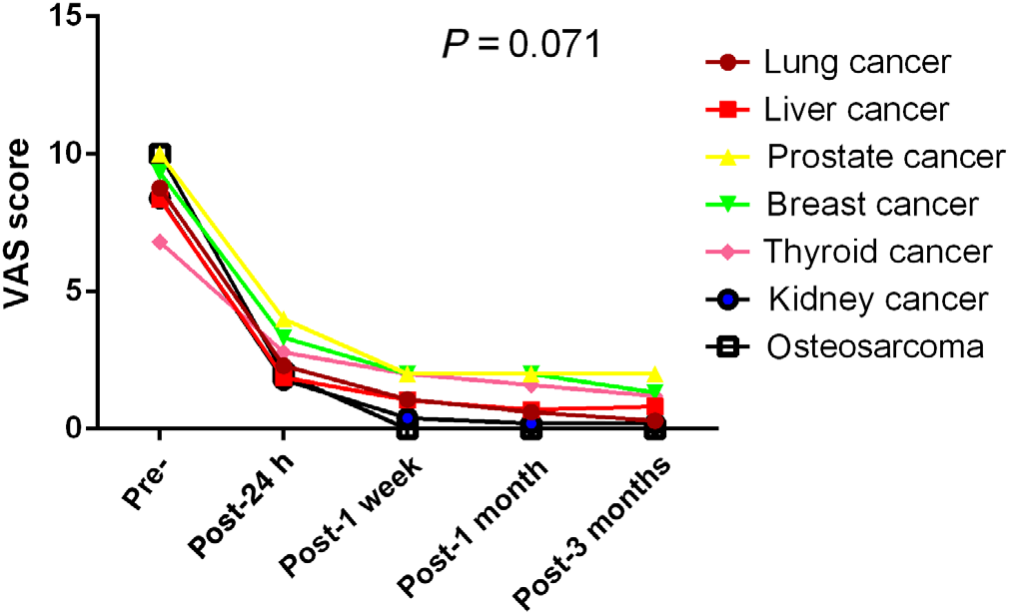
Changes in visual analogue scores from baseline to 3 months among different primary lesions.

(3) Whether there are any differences between the three image‐guided percutaneous microwave ablation procedures for treating painful osseous metastases: No significant differences in VAS scores were observed among the different imaging techniques and ablation energies. In patients who also had soft tissue masses, there was no difference in the VAS scores between CT and fluoroscopy with ultrasound. The VAS score curves are shown in Figs 8, 9, 10, 11.

## Discussion

Bone metastasis is common and occurs in approximately 20% of patients with cancer^24^, ^27^. These metastases stem from the prostate or breasts in 70% of cases, the lungs or kidneys in 20%–30% of cases, and the rectum, colon, ovaries, and pancreas in 10% of cases^2^. The majority (66%) of bone metastases are extra‐spinal, occurring mostly in the pelvis and sacrum^28^, ^29^. Skeletal metastatic disease can cause various complications, including pain, pathologic fractures, declining mobility, deconditioning, weakness, decreased respiratory function, and metabolic disorders, such as severe hypercalcemia^30^. Due to short life expectancies, treatment strategies tend to be palliative rather than curative^3^.

### *Pain Relief*

Some studies that have used MWA to treat patients with painful metastases have demonstrated promising results^31^, ^32^. Khan *et al*.^4^ reported immediate pain reduction in 94% (65/69) of these procedures, which was maintained for 6 months. However, the mechanism of pain relief remains unclear. This may include destruction of the nerve fibers for pain in the periosteum and bone cortex, reduction in the burden of the tumor with reduced transmission of pain *via* the nerve endings, decreased osteoclastic activity and coagulative necrosis of the tumor cells, and resultant decrease in the production of nerve‐stimulating cytokines such as interleukins and tumor necrosis factor^31^. Our results suggest that all patients experienced pain relief on postoperative day 2, with no pain recurrence at the ablation site during the 3‐month follow‐up period. Only 1 patient died during the follow‐up period: an individual with metastatic liver cancer who died of multiorgan dysfunction syndrome 3 months post‐surgery but without the recurrence of pain. Therefore, percutaneous MWA ensured significant and lasting pain relief and improved the end‐of‐life care for these patients with end‐stage neoplasms and intractable pain.

### *Choice of Various Image‐Guided Procedures*

Percutaneous MWA can be currently performed under imaging guidance. Fluoroscopy‐guided percutaneous ablation is suitable for combination with vertebroplasty due to its low resolution of soft tissue^23^; Khan *et al*.^4^ published the largest study series of microwave ablation and cement vertebroplasty, treating 102 lesions in 69 patients. Evidence for the safety and efficacy of percutaneous ablation has been demonstrated. In the current study, fluoroscopic guidance was selected in 23 cases. All the lesions were within the vertebral body, and vertebroplasty was performed immediately after ablation of the lesion so that positioning could be achieved with simple fluoroscopic guidance. Westbroek reported a case performed under fluoroscopy, with the resultant complication of thermal injury to the spinal cord^33^.

CT‐guided procedures^34^ are widely used as they enable accurate localization and are suitable for any site, including complex sites such as the pelvis and spine^28^, ^29^. In the current study, CT guidance was used in 16 patients with lesions near the pelvis and ribs. The anatomy of these sites is relatively complicated, with many delicate organs in the vicinity. CT can provide precise positioning but requires a large operational space, is time‐consuming, and has some radiation effects on patients and surgeons. Therefore, Burgard *et al*. introduced low‐milliampere CT fluoroscopy during CT‐guided ablation^34^ to reduce the adverse effects associated with radiation. However, microscopic foci of residual disease cannot be identified with standard imaging^4^; furthermore, we believe that CT imaging cannot reflect the extent and size of ablation in real time and that guidance is only available to the extent of parameters provided by the manufacturer's data specification and our operational experience.

To overcome these shortcomings, we performed selected fluoroscopy combined with ultrasound‐guided surgery in cases of osseous metastasis with soft tissue masses. Although fluoroscopic positioning is not precise, it produces little radiation. Ultrasound has relatively poor accuracy in bone tissue detection and is generally used in soft tissue tumor ablation; however, it provides real‐time visualization of applicator placement and is both universally available and of low cost^35^, ^36^. These two techniques were combined in the present study of 11 patients with osseous metastasis of soft tissue masses. Because tumor tissue destroys the cortical bone, positioning of the ablation needle and change in tumor echogenicity due to high temperatures could be observed in real time under ultrasound guidance^37^. Compared to CT and fluoroscopy alone, the combined thermal monitoring technique provides real‐time intraoperative monitoring of the extent of ablation^35^. However, this operation is only suitable for single MWA needle treatment, which is consistent with the findings of many researchers^23^. Ultrasound provides a two‐dimensional image, which affects the accuracy of ablation, and the gas produced by MWA prevents accurate intra‐procedural monitoring of the ablation zone^23^. Although MRI has considerable advantages in guiding percutaneous ablation of metastases^38^, we did not have the equipment to perform this procedure in our MRI facility.

### *Structural Stabilization*

Osteolytic metastases can cause pathological fractures, and an impending fracture should be treated before the fracture occurs^39^. Cementoplasty is performed with a palliative intent and does not stop tumor progression; it treats pain and allows fast consolidation of weight‐bearing bones^40^, such as the vertebrae and acetabula. MWA alone can cause local instability as a direct result of therapeutic effects or tumor regression. This phenomenon is similar to increased risk of pathologic fractures after radiotherapy, which has a reported incidence of 15%–40%^41^, ^42^, ^43^. We used bone cement for ablation of osseous lesions, as reported elsewhere^44^. Thus, there were 14 cases of MWA combined with bone cement in this study. No pathological fractures were found during the 3‐month follow‐up period, and the effect of pain relief was higher due to the reinforcement of osteolytic destruction^4^, ^45^. Our results demonstrate that a combination of MWA with cementoplasty is an effective palliative treatment for intractable pain and for preventing pathologic fractures.

### *Functional Activity*

With respect to the functional evaluation of this treatment, most patients exhibited improved functional performance. Pusceddu reported that 98% of patients with large lesions obtained overall functional improvement 6 months after the procedure. Only 1 patient experienced a worsened functional outcome^24^. In our study, all patients demonstrated significant functional improvement during the 3‐month follow‐up period. Due to the improvement in pain and reinforcement with bone cement, instability of the load‐bearing bones was avoided.

### *Choice of Anesthesia*

Most researchers have suggested sedation combined with local anesthesia^15^, ^40^, ^46^. In our opinion, for deep tissue ablation (such as of the spine and iliac bone), local anesthesia alone can be sufficient, as seen in 23 cases in the present study. For superficial tissue ablation (such as rib and ischium) and concurrent treatment of multiple sites, general anesthesia or sedation with local anesthesia is recommended.

### *Limitations*

The present study has some limitations. First, this study had no control group and only a small number of cases were included. Second, this is a retrospective analysis from only one institution and the data might be limited by referral bias.

## Conclusion

Image‐guided percutaneous MWA of osseous metastasis significantly relieved pain and improved mobility; however, the decision to use a combination of bone cement in weight‐bearing bones should be taken on a case‐by‐case basis. CT is the preferred imaging modality for percutaneous ablation, while fluoroscopy combined with ultrasound is effective for cases with soft tissue masses. The hyperechoic area arising during the operation and the thermal monitoring system provide real‐time intraoperative monitoring of the extent of ablation. Fluoroscopy is suitable for combination with vertebroplasty. Further research with larger study populations is needed to assess the utility of image‐guided percutaneous MWA and to validate the findings of this study.
